# Recurrence Rate Following Breast Conservation Surgery: A Retrospective Cohort Study

**DOI:** 10.1155/tbj/5063047

**Published:** 2026-03-12

**Authors:** Liyang Wang, Yuchen Yuan, Urvashi Jain, Eleftheria Kleidi

**Affiliations:** ^1^ Cambridge Breast Unit, Cambridge University Hospitals NHS Foundation Trust, Cambridge, UK, cuh.org.uk; ^2^ Department of Health Services Research & Policy, London School of Hygiene & Tropical Medicine, London, UK, lshtm.ac.uk; ^3^ School of Clinical Medicine, University of Cambridge, Cambridge, UK, cam.ac.uk

**Keywords:** breast-conserving surgery, IBTR, margins, recurrence, survival

## Abstract

**Background:**

Breast‐conserving surgery (BCS) has become the standard treatment for early‐stage breast cancer, offering oncological safety, better breast aesthetics, and enhanced health‐related quality of life. The optimal margins for BCS have been debated. However, evidence on the impact of margin widths greater than 0 mm for invasive breast cancer remains limited. Cambridge Breast Unit (CBU) has adopted a policy where a single margin of < 1 mm (not at ink) for invasive disease would be acceptable, provided the other three radial margins are ≥ 1 mm.

**Method:**

Retrospective cohort study analyzed 372 women who underwent BCS for invasive breast cancer at CBU between 2015 and 2016. Patients with pure ductal carcinoma in situ (DCIS), prior breast cancer treatment, or neoadjuvant chemotherapy were excluded. Clinical data, including patient demographics, tumor characteristics, margin status, and recurrence and survival outcomes, were extracted from electronic records.

**Results:**

At a median follow‐up of 5.2 years, the ipsilateral breast tumor recurrence (IBTR) rate was 1.6% (6/372) with an overall recurrence rate of 5.6% (21/372). No significant association was found between final resection margins and local or overall recurrence rates, nor was margin status correlated with breast cancer recurrence‐free survival. A total of 53 patients (14.2%) required additional surgeries due to margin status. By accepting a single radial margin < 1 mm, our margin policy reduced reoperations by 33%.

**Conclusion:**

Margin assessment in breast surgery is multifaceted, requiring a personalized approach that considers tumor biology, systemic treatments, and pathological variability. Our findings support accepting a margin width of < 1 mm (no tumor on ink) for a single radial margin in invasive breast cancer, provided the other three radial margins are ≥ 1 mm. It reduces reoperations without compromising oncological outcomes. Integrating advanced intraoperative techniques and multidisciplinary decision‐making will further optimize patient care and long‐term outcomes.

## 1. Background

Breast‐conserving surgery (BCS) followed by radiotherapy (RT) emerged in the 1970s and has since become the standard treatment for the majority of early‐stage breast cancers. BCS not only maintains breast aesthetics but also enhances health‐related quality of life (HR‐QOL). Additionally, it has demonstrated oncological safety, with survival rates and recurrence rates comparable to those of mastectomy [1–3].

Resection margins significantly influence local recurrence (LR) rates following BCS, and the optimal margin has been a contentious issue over the years. Large‐scale meta‐analyses have shown that ink on the tumor is linked to a higher incidence of ipsilateral breast tumor recurrence (IBTR) compared to cases with no tumor at the inked margin for invasive breast cancer [4]. Specifically, positive margins result in an odds ratio of 2.44 for tumor recurrence, while close margins yield an odds ratio of 1.74 [4]. This evidence supports the 2014 consensus from the Society of Surgical Oncology and the American Society for Radiation Oncology, which recommends adopting “no ink on tumor” as the standard for surgical margins for invasive breast cancers [5]. However, there is a lack of sufficient data on margins greater than 0 mm and the updated meta‐analysis also demonstrated that an increase in margin width from no tumor on ink to 1 mm, 2 mm, or 5 mm had no influence on LR with adjustment for follow‐up time [4]. Therefore, the definition of “no tumor on ink” was widely accepted as the negative margins’ definition for invasive breast cancers across the globe.

The considerations for margin width following surgical excision of ductal carcinoma in situ (DCIS) are distinct from those for invasive carcinoma, primarily because DCIS often displays a discontinuous growth pattern that requires careful assessment for adequate surgical clearance.

In 2015, the Association of Breast Surgery (ABS) in the UK voted to recommend a 1‐mm margin for invasive, pure DCIS and invasive disease admixed with DCIS based on a small number of studies with comparison of tumor on ink versus 1 mm and the nuances of pathological examination [6].

Following review of evidence, and with an aim to reduce unnecessary reoperations which could significantly hamper the cosmesis without achieving a benefit in oncological outcomes, the Cambridge Breast Unit (CBU) updated the unit policy for margins in 2014: For invasive carcinoma, with or without DCIS, a single margin of < 1 mm (not at ink) would be acceptable, provided that there was no tumor at ink and no evidence of disease at multiple close radial margins. This study aims to evaluate the efficacy of the change in the unit margins’ policy, specifically the association of margin status on the local and overall recurrence rate of breast cancer after 5 years of follow‐up.

## 2. Methods

In this retrospective cohort study, we included women who underwent breast‐conserving therapy (BCT—BCS and RT) for invasive cancer at CBU, Addenbrooke’s Hospital, in a 2‐year period between January 1, 2015, and December 31, 2016. Exclusion criteria included pregnancy, bilateral breast cancer, previously treated breast cancer, patients who received neoadjuvant chemotherapy, and patients who were lost to follow‐up. Patients having BCS for pure DCIS were also excluded from the analysis. As RT forms an essential part of BCT to reduce recurrence and breast cancer–related mortality [7], we excluded patients who failed to receive RT following BCS, unless they had subsequently undergone completion mastectomy. Relevant clinical information was collected from electronic medical records. This included patient demographics, tumor histology, final radial margin status (after re‐excision when applicable), and any adjuvant treatments received.

At our institution, the wide local excision specimen is oriented by the operating surgeon at the time of surgery; the superior, anterior, and medial margins are specifically marked using either clips or sutures to enable anatomical correlation. Margin width is assessed for the four radial margins (superior, inferior, medial, and lateral) and the anterior and posterior margins on the main specimen. Where cavity shave margins are taken, these are also oriented with the cavity side marked, and their assessment is considered alongside the corresponding radial margin(s) where applicable. Following receipt in the laboratory, specimens are handled and inked in the pathology department by pathology staff, and margin distances are recorded on histological assessment.

During the study period (2015–2016), there were no changes to specimen orientation, inking practice, or overall specimen handling, and the approach used was the same as in the preceding years. Prior to the adoption of the revised margin policy, local practice followed contemporaneous ABS guidance, recommending re‐excision where the radial margin was < 2 mm [6]. Under the revised policy, a “single close margin < 1 mm (not at ink)” was defined as only one radial margin measuring < 1 mm, with the other three radial margins ≥ 1 mm. Re‐excision was not routinely recommended in our MDT for a single close margin < 1 mm provided there was no tumor on ink and no other multifocal close radial margins. However, where two or more radial margins were < 1 mm (i.e., multifocal close margins), re‐excision was generally advised.

All patients were discussed at the breast cancer MDT. All women who had BCS received adjuvant RT to the breast as per the unit RT policy. Endocrine therapies were prescribed based on receptor status and patient tolerance. Chemotherapy and targeted treatments were recommended to those patients with predicted high benefit as per MDT discussion.

At the CBU, patients are routinely followed up with yearly mammograms for at least 5 years. They are discharged to patient‐led follow‐up and may present to the breast unit at any time should any concerns arise. A clinical evaluation would take place and investigations would be requested as necessary in these situations. For this study, follow‐up time was calculated from the date of the surgery to the date of their most recent mammogram or cross‐sectional imaging scan (i.e., CT thorax/MRI breast/PET scan). Follow‐up data were collected up to December 2022. LRs were defined as ipsilateral in‐breast tumor recurrences. Axillary recurrences were categorized as regional recurrences. Cancer spread to distant sites was categorized as distant recurrence.

IBM SPSS Version 26.0 (IBM, US) and Stata MP 17.0 (StataCorp, US) were used for statistical analysis, and *p* < 0.05 was considered statistically significant.

This study has been approved by Cambridge University Hospitals NHS Foundation Trust (Register number: PRN9497). All patient data are presented in anonymized format, and ethical approval was determined to be unnecessary.

## 3. Results

### 3.1. Patient and Tumor Characteristics

Between 2015 and 2016, a total of 553 patients underwent BCS at the CBU. Following application of exclusion criteria, 372 women were included in the analysis. Overall recurrence rate at 5 years was 5.6% (21/372 patients).

The patient and tumor characteristics can be found in Table 1. The age of the patients included in the study ranged from 28 to 97 years, and the mean age at operation was 62.8 years. Out of these women, 162 presented symptomatically, while 209 had screen‐detected malignancy and a further 1 patient had incidental breast cancer identified while having imaging for other conditions. The median follow‐up time was 5.2 years (interquartile range 5.0–5.8 years).

**TABLE 1:** Patient and tumor characteristics.

		Total cohort N = 372	Any recurrence *n* = 21		*p* (any recurrence)^∗^	IBTR *n* = 6		*p* (IBTR)^∗^
			Number	Row %		Number	Row %	
Age (Mean ± SD)	—	62.8 ± 11.2	66.8 ± 10.4	—	0.0924 (Student *T* test)	64.6±10.5	—	0.828 (Student *T* test)
Method of presentation	Screen detected	209	7	3.3	0.094	4	1.9	0.705
	Symptomatic	162	14	8.6	—	2	1.2	—
	Incidental finding	1	0	0	—	0	0	—

Histology	NST	262	17	6.5	0.849	5	1.9	0.748
	ILC	47	2	4.3	—	0	0	—
	Invasive tubular	11	0	0	—	0	0	—
	Mixed	30	2	6.7	—	1	3.3	—
	Other	22	0	0	—	0	0	—

Invasive disease with DCIS	Yes	241	16	6.6	0.349	6	2.5	0.094
	No	131	5	3.8	—	0	0	—

Estrogen receptor	Positive	344	4	1.2	0.063	4	1.2	0.068
	Negative	28	17	60.7	—	2	7.1	—

HER2 receptor	Positive	25	16	64	0.014	3	12	0.010
	Negative	342	5	1.5	—	3	0.9	—
	Not tested	5	0	0	—	0	0	—

Grade	1	77	2	2.6	0.431	1	1.3	0.514
	2	193	11	5.7	—	2	1	—
	3	98	8	8.2	—	3	3.1	—
	Unclear	4	0	0	—	0	0	

T stage	T1	218	10	4.6	0.224	4	1.8	1.000
	T2	146	10	6.8	—	2	1.4	—
	T3	8	1	12.5	—	0	0	—

N stage	N0	292	13	4.5	0.001	5	1.7	1.000
	N1	62	4	6.5	—	1	1.6	—
	N2	4	1	25	—	0	0	—
	N3	4	3	75	—	0	0	—
	Nx	10	0	0	—	0	0	—

Tumor extent	Multiple invasive foci	57	17	29.8	0.544	0	0	0.596
	Localized	315	4	1.3	—	6	1.9	—

Lymphovascular invasion	Yes	64	12	18.8	0.014	2	3.1	0.439
	No	294	9	3.1	—	4	1.4	—
	Unclear	14	0	0	—	0	0	—

^∗^Fisher’s exact test unless otherwise specified.

A majority (70.4%) of patients (262/372) had no specific type (NST) of breast cancer. The second most common type of cancer was invasive lobular cancer in 12.6% of patients (47/372), followed by invasive tubular cancer in 3.0% of patients (11/372). In the postoperative histology, 77 (20.7%) patients had Grade 1 tumor, 193 (51.9%) had Grade 2 tumor, and 98 (26.3%) had Grade 3 tumor. In 4 patients, tumor grade could not be assessed due to extremely small tumor size. A total of 241 patients (64.8%) had DCIS accompanying the invasive tumor. Most patients (92.5%, 344/372) had ER‐positive tumors, and a small proportion of patients (6.7%, 25/372) had HER2‐positive tumors.

### 3.2. Resection Margins

Patients were categorized into three groups based on their final radial excision margins, taking into account any re‐excisions: *involved* (tumor at the ink), *close* (< 1 mm but no tumor at the ink), and *wide* (> 1 mm). Of the total cohort, 14 patients (3.8%) had involved final margins, despite adherence to unit policy. This outcome was primarily due to patient‐specific factors (such as preference, surgical fitness, or death prior to surgery) or acceptance of the margins following multidisciplinary team (MDT) discussion, where no further tissue was available for resection in the radial direction. Additionally, 26 patients (7.0%) had close margins and, in accordance with policy, were not subjected to further surgery.

### 3.3. Reoperation and Systemic Treatments

A total of 53 patients (14.2%) required additional surgeries due to involved or close margins. Among these, 10 patients underwent more than one reoperation to achieve an adequate margin. A majority of these patients (64.2%, 34/53) underwent further re‐excision of margins, while 19 patients (35.8%) ultimately had completion mastectomies. A detailed breakdown of patient treatments and clinical outcomes is provided in Table 2.

**TABLE 2:** Reoperation rates and systemic treatments.

		Total cohort N = 372 (%)	Any recurrence (*n* = 21) (%)	*p* (any recurrence)^∗∗^	IBTR (*n* = 6) (%)	*p* (IBTR)^∗∗^
Reoperation	Any reoperation	53 (14.2)	1 (4.8)	0.648	0	1.000
	Re‐excision	34 (9.1)	1 (4.8)	—	0	—
	Completion mastectomy	19 (5.1)	0	—	0	—

Final margin status	Involved (tumor at ink)	14 (3.8)	1 (4.8)	0.597	1 (16.7)	0.128
	Close (< 1 mm but no tumor at ink)	26 (7.0)	2 (9.5)	—	1 (16.7)	—
	Wide (> 1 mm)	332 (89.2)	18 (85.7)	—	4 (66.7)	—

Endocrine therapy	Yes	288 (77.4)	10 (47.6)	0.012	2 (33.3)	0.025
	No	84 (22.6)	11 (52.4)	—	4 (66.7)	—

Adjuvant chemotherapy	Yes	72 (19.4)	15 (71.4)	0.263	2 (33.3)	0.329
	No	300 (80.6)	6 (28.6)	—	4 (66.7)	—

Radiotherapy	Yes	360 (96.8)	21 (100)	1.000	6 (100)	1.000
	No	12 (3.2)	0	—	0	—

Recurrence	Local	6 (1.6)^∗^	6 (28.6)^∗^	—	6	—
	Regional	3 (0.8)^∗^	3 (14.3)^∗^	—	—	—
	Distant	14 (3.8)^∗^	14 (66.7)^∗^	—	1 (16.7)^∗^	—
	All recurrences	21 (5.6)	—	—	—	—
	No	351 (94.4)	—	—	—	—

^∗^Some patients had both local/regional and local/distant recurrences.

^∗∗^Fisher’s exact test unless otherwise specified.

### 3.4. Breast Cancer Recurrence

During the follow‐up period, 5.6% (21/372) of patients developed either local, regional, or distant recurrences. Of these, 6 patients had IBTR, giving an IBTR rate of 1.6% for our patient cohort. Among these patients with IBTR, the median time to recurrence was 4.1 years.

The comparison of patient and tumor characteristics and overall recurrence is presented in Tables 1 and 2. There was no significant association between recurrence and factors such as age, mode of presentation, histology, in situ component, grade, T stage, or tumor extent. Similarly, reoperation, final resection margin, adjuvant chemotherapy, or RT did not show a statistically significant association with tumor recurrence. However, HER2 receptor status and endocrine therapy were significantly associated with recurrence outcomes. Despite 344 patients having ER‐positive tumors, only 288 (83.7%) ultimately received endocrine therapy; due to intolerance, comorbidities, or an MDT assessment deemed them to derive minimal survival benefit. Patients with HER2‐positive tumors were found to be 5 times more likely to develop recurrence compared to those that were HER2‐negative (OR 5.09, *p* = 0.004, 95% CI 1.69–15.32). Additionally, N stage and lymphovascular invasion were also associated with an increased risk of overall breast cancer recurrence. Specifically, patients with N3 disease had an odds ratio of 64.4 (*p* < 0.001, 95% CI 6.36–662.07) for developing overall recurrence compared to N0.

Similar results were observed when comparing patients with local recurrences to those without. Statistically significant correlations were found with HER2 receptor status, as well as with endocrine therapy. On univariable analysis, HER2 positivity was associated with higher odds of IBTR (OR 15.4, 95% CI 2.93–80.3), while patients who received endocrine therapy were associated with lower odds of IBTR (OR 0.14, 95% CI 0.025–0.78)^1^. However, lymphovascular invasion and N stage did not appear to be significant factors. Notably, resection margins did not have a significant impact on IBTR (*p* = 0.128).

Kaplan–Meier analysis of breast cancer recurrence‐free survival based on different final resection margin statuses is shown in Figure 1. The log‐rank test confirmed no significant association (*p* = 0.72). A second analysis was conducted to specifically examine local recurrence‐free survival. Although Kaplan–Meier analysis appears to have a possible trend, it is worth noting that the sample size is very small (*N* = 6). The log‐rank test did not show statistical significance (*p* = 0.08).

FIGURE 1Kaplan–Meier recurrence‐free survival. (a) All recurrence‐free survival and (b) local recurrence‐free survival.

**Figure:**
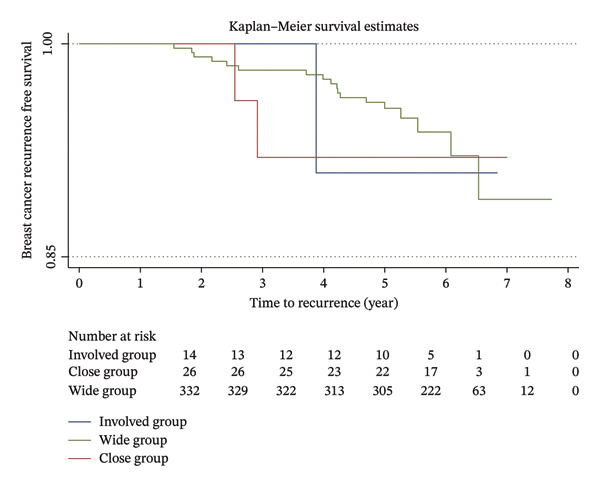
(a)

**Figure:**
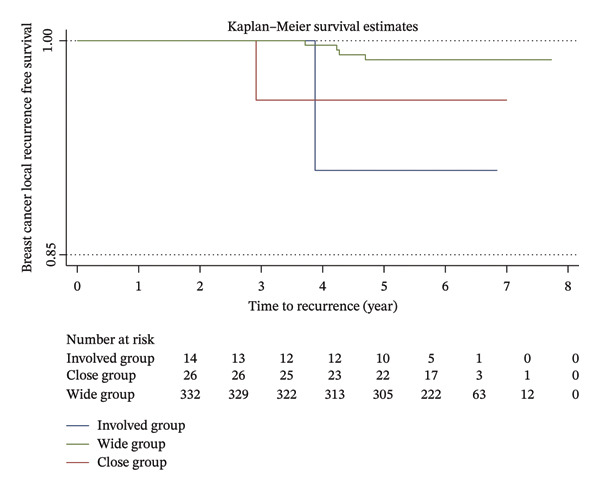
(b)

## 4. Discussion

The optimal or adequate margin after BCS has always been a matter of debate and controversy. Although involved or close margins have been shown to be associated with an increased rate of IBTR [4], there has not been clear evidence on the impact of margins on regional and/or distant recurrences and overall survival. In this single‐center retrospective cohort study, we included 372 women who underwent primary BCT for a new diagnosis of unilateral invasive breast cancer between 2015 and 2016.

The prevalence of IBTR in our patient cohort was 1.6% at a median follow‐up of 5.2 years. Our IBTR rate is low and in accordance with ESMO recommendations of < 0.5% per year and < 5% over 5 years [8]. Our patient cohort had a locoregional recurrence rate of 2.1% (8/372) and a distant recurrence rate of 3.8% (14/372). These were comparable to a recent registry‐based study from Germany, where they reported 2.1% of locoregional recurrence and 3.0% of distant recurrences at 5 years in patients receiving BCT [9].

To contextualize our results against our own historical outcomes, an earlier paper from our unit of patients undergoing BCS between 1999 and 2004 reported a very low LR of 1.1% using a 5‐mm target radial margin with a median follow‐up of 58 months [10]. In our contemporary cohort, our IBTR remained low (1.6%). However, these cohorts are not directly comparable as many aspects of breast cancer care have evolved substantially over time, particularly systemic therapy and RT practice. Given these limitations, our data suggest that adopting the current margin policy has not been associated with any obvious deterioration in local control.

With regard to reoperation rate, 14.2% of patients underwent re‐excision and/or mastectomy from our cohort. With the implementation of the new margin policy, we spared 26 patients (7.0%) from additional surgery, reducing the reoperation rate by 33%. In comparison, the England national study using hospital episode statistics in 2012 showed a reoperation rate of 20% [11], putting our center well below the national average. Our reoperation rate is also favorable compared to data reported internationally, such as from the United States of America (23% out of 2206 patients) [12], Germany (21.4% of 565 patients) [13], and the Netherlands (28.9% of 961 patients) [14]. This indicates that our approach reduces the need for reoperation (which could have a detrimental effect on cosmesis) while achieving good oncological outcomes.

In a recent meta‐analysis, a significant increase in both local and distant recurrence rates was reported with involved or close pathological margins, with hazard ratios of 1.98 and 2.10, respectively [15]. However, it has been suggested that the close margin defined in the meta‐analysis (< 2 mm) only has a weak effect on survival and may be confounded by bias. Additionally, the conclusive recommendation of a minimum margin width of 1 mm made by the authors is a pragmatic decision rather than an evidence‐based study‐led conclusion. In our cohort, we found no statistically significant association between margin status and local, regional, or distant recurrences. Although there appears to be a trend of increased IBTR rate in patients with close or involved margins from the Kaplan–Meier analysis, these were not statistically significant. This is likely due to the very small absolute numbers of IBTR we observed in our patient cohort (6 patients in total). Further analysis with a larger patient population and longer follow‐up would be necessary to confirm these findings.

Multiple other factors are reported to impact the rate of local, regional, and distant recurrence following breast cancer treatment. These include patient age [16], tumor characteristics (size, type, grade, receptor status, multifocal or unifocal, lymphovascular invasion) [17], nodal status, and any adjuvant treatment received [18, 19]. Although our study confirmed the association of certain tumor factors with higher rates of recurrence, it is difficult to recruit a large enough patient cohort stratified by tumor biology and other factors to determine the effect on margin status.

There are patients in our cohort who did not receive endocrine treatment despite having ER‐positive disease. The decision was made collaboratively by physicians considering factors such as comorbidities, frailty, impact on survival, and the patient’s ability to tolerate the treatment. The absence of endocrine therapy was linked to an increased risk of recurrence, which aligns with established evidence that endocrine therapy significantly reduces recurrence rates and breast cancer mortality in ER‐positive patients [20].

We found that patients with HER2‐positive disease had a higher risk of IBTR and overall recurrence, consistent with findings from large cohort studies [16, 17]. Additionally, HER2‐positive cancers are often associated with early recurrences within the first 5 years of diagnosis [21]. Moreover, the study cohort was from a time (during the Years 2015 and 2016), prior to the approval of dual blockade with pertuzumab for patients with HER2‐positive disease in the UK [22]. This may have contributed to a higher recurrence rate in this group, demonstrating the effect of systemic treatment on breast cancer recurrence.

Moreover, we observed that heavy nodal involvement (especially N3 disease) is associated with an increased overall recurrence rate but not IBTR. This is again an expected finding as nodal involvement is well established as a predictor of regional (axillary) and distant recurrences, as well as poor survival [23, 24].

### 4.1. Limitations

This retrospective study was conducted at a single hospital, which, while limiting the size and diversity of the patient cohort, allowed us to have greater consistency in the local policies, including pathology analysis, margin strategy, and adjuvant treatment approaches. The observational study design invariably made it difficult to fully account for potential bias and confounding factors. However, the uniformity in institutional practices assisted in limiting the effects of those factors on our findings.

While we have explored the intricacies of tumor biology and patient characteristics, it is crucial to acknowledge the variability inherent in pathological assessments of specimens. This variability significantly influences margin evaluation and reporting [25]. Moreover, the advent of advanced intraoperative techniques and technologies holds promise for transforming this aspect of surgical practice in the future [26].

A further limitation is that a major upgrade of local electronic systems in 2014 limited complete case ascertainment/linkage for the preceding years, preventing a continuous local pre/post‐time‐trend comparison. In addition, given that 92% of the cohort was ER‐positive, a median follow‐up of 5 years is relatively short for fully capturing late events. Nonetheless, this study contributes real‐world evidence from a contemporary cohort, adding to the wider evidence base and supporting future pooled analyses and meta‐analyses of margin policy and reoperation outcomes.

From a future viewpoint, studies like SMALL are pioneering vacuum‐assisted excisions without a mandated margin for patients with small tumors [27]. This approach signifies a shift away from a one‐size‐fits‐all approach regarding margins. Instead, a more nuanced decision‐making process within the MDT is essential.

## 5. Conclusion

In our single‐center patient cohort, we did not find a significant association between the final radial margin and the incidence of local or overall recurrence of breast cancer. However, we found that HER2 positivity and failure to receive endocrine therapy are associated with an increased risk of overall recurrence rates, confirming the crucial role of tumor biology and systemic treatments on breast cancer recurrence. Additionally, heavy nodal involvement is associated with an increased overall recurrence rate. By adopting a single final radial margin width of < 1 mm (with no tumor on ink), while ensuring all the other radial margins were ≥ 1 mm, we successfully reduced the reoperation rate by 33%. To validate the reproducibility of our results, we plan to expand the patient cohort and extend the follow‐up period in future studies.

In conclusion, it is imperative to recognize the multifaceted nature of margin assessment in breast surgery. The integration of advanced intraoperative techniques, coupled with a tailored approach to margin management with a good understanding of various other factors such as variability in pathological assessment of specimen, tumor biology, advanced systemic treatments, and their effects, can significantly enhance patient outcomes. As we continue to refine our strategies, we must prioritize collaboration within the MDT to ensure that decisions reflect a balance between oncological safety and the preservation of breast aesthetics. This holistic approach will not only improve clinical efficacy but also enhance the overall quality of life for our patients.

## Funding

No funding was received for this research.

## Conflicts of Interest

The authors declare no conflicts of interest.

## Endnotes

^1^Estimates are based on a small number of IBTR events (*n *= 6); odds ratios and confidence intervals are therefore imprecise and may be sensitive to sparse data/separation.

## Data Availability

The data that support the findings of this study are available upon request from the corresponding author. The data are not publicly available due to privacy or ethical restrictions.
